# Nano-Structured Materials under Irradiation: Oxide Dispersion-Strengthened Steels

**DOI:** 10.3390/nano11102590

**Published:** 2021-10-01

**Authors:** Joël Ribis, Isabelle Mouton, Cédric Baumier, Aurélie Gentils, Marie Loyer-Prost, Laurence Lunéville, David Siméone

**Affiliations:** 1 Université Paris Saclay, CEA, Service de Recherches Métallurgiques Appliquées, 91191 Gif-sur-Yvette, France; isabelle.mouton@cea.fr (I.M.); laurence.luneville@cea.fr (L.L.); david.simeone@cea.fr (D.S.); 2 Université Paris-Saclay, CNRS/IN2P3, IJCLab, 91405 Orsay, France; cedric.baumier@in2p3.fr (C.B.); aurelie.gentils@ijclab.in2p3.fr (A.G.); 3 Université Paris Saclay, CEA, Service de Recherches de Métallurgiques Physiques, 91191 Gif-sur-Yvette, France; marie.loyer-prost@cea.fr

**Keywords:** nuclear material, nano-oxides, ion irradiation, Ostwald ripening, stability, transmission electron microscopy

## Abstract

Oxide dispersion-strengthened materials are reinforced by a (Y, Ti, O) nano-oxide dispersion and thus can be considered as nanostructured materials. In this alloy, most of the nanoprecipitates are (Y, Ti, O) nano-oxides exhibiting a Y_2_Ti_2_O_7_ pyrochlore-like structure. However, the lattice structure of the smallest oxides is difficult to determine, but it is likely to be close to the atomic structure of the host matrix. Designed to serve in extreme environments—i.e., a nuclear power plant—the challenge for ODS steels is to preserve the nano-oxide dispersion under irradiation in order to maintain the excellent creep properties of the alloy in the reactor. Under irradiation, the nano-oxides exhibit different behaviour as a function of the temperature. At low temperature, the nano-oxides tend to dissolve owing to the frequent ballistic ejection of the solute atoms. At medium temperature, the thermal diffusion balances the ballistic dissolution, and the nano-oxides display an apparent stability. At high temperature, the nano-oxides start to coarsen, resulting in an increase in their size and a decrease in their number density. If the small nano-oxides coarsen through a radiation-enhanced Ostwald ripening mechanism, some large oxides disappear to the benefit of the small ones through a radiation-induced inverse Ostwald ripening. In conclusion, it is suggested that, under irradiation, the nano-oxide dispersion prevails over dislocations, grain boundaries and free surfaces to remove the point defects created by irradiation.

## 1. Introduction

Nanostructured materials, ranging from nanocrystalline and nanolayer to nanoporous and nanocomposite structures, are materials whose structural elements have dimensions in the range of 1–100 nm [1]. Over the past decade, an explosion in both academic and industrial interest in these materials has arisen due to the remarkable and unique properties—such as fundamental electric, optical, magnetic and mechanical properties—that they exhibit in various fields [1]. Oxide dispersion-strengthened steels can be considered to be examples of nanostructured materials owing to the high density of nano-oxides embedded in the ferritic matrix. The nano-oxides essentially serve as obstacles for dislocation motion and confer excellent mechanical strength properties to the alloy. Thus, these alloys are good contenders for applications in harsh environments as core component materials for the future generation of nuclear power plants. Therefore, maintaining this precipitation at a nanoscale in service conditions is of great interest to preserve the excellent mechanical properties and to guarantee the safe use of reactors. However, nanostructured ODS materials are far from equilibrium due to the high density of precipitate/matrix interfaces. Thus, the associated contribution of these interfaces to free energy constitutes a large driving force that leads the nano-oxides to coarsen even under irradiation. Therefore, to keep the benefits of nanoprecipitation by preventing the nano-oxides from coarsening, their irradiation stability has to be thoroughly understood in extreme conditions. Thus far, a number of studies have investigated the irradiation evolution of oxide nanoparticles in *bcc* Fe-Cr-based oxide dispersion-strengthened steels [2]. The main contributing mechanisms for nanoparticle evolution have been identified [2,3]: ballistic dissolution, Ostwald ripening, irradiation-enhanced diffusion and homogeneous nucleation. Of these four major themes explaining the irradiation response of the nanoparticles, this paper develops the two first themes.

- Ballistic dissolution refers to the displacement of a knock-on-atom to a position in the surrounding matrix outside the nano-oxide [2]. An interfacial atom must be ejected more than one nearest-neighbour distance in the adjacent matrix to separate itself from the nano-oxide [2]. The result is the total or partial dissolution of the nano-oxide dispersion. He et al. [4] observed a significant decrease in size and number density of the nano-oxide distribution after 5 MeV Ni^2+^ irradiation at 300 °C for a damage level of 100 dpa. If this phenomenon is normally expected at low temperature, some authors also reported dissolution at higher temperature. Li et al. [5] observed nanoparticle shrinking under electron irradiation at 400 °C in a Fe-9Cr ODS. Swenson and Wharry [6] observed the dissolution of the nano-oxides in Fe-9Cr ODS steels after neutron irradiation up to 3 dpa at the even higher temperature of 500 °C.
- Ostwald ripening under irradiation is similar to the well-known thermal process where small particles shrink to the benefit of large ones to minimize interfacial energy; the main difference is the irradiation cascades, which help in increasing interfacial solute concentration and enhance solute transport [3]. Ostwald ripening can also be inverted, leading the small particles to grow at the expense of the large ones [3]. In ODS steels, similar to those studied in this paper, Lescoat et al. [7] proved that after irradiation, the average size of the nano-oxides increased while their density decreased. They show that the radius evolution kinetics of the nano-oxides can be scaled as *t*^1/3^, with *t* being the irradiation time, while the density decreased linearly with the inverse of the irradiation time, which is conformed to a classic Ostwald ripening process [8]. Ostwald ripening was also reported in other studies [9,10]. Chen et al. [11] distinguished the coarsening behaviour of coherent nano-oxides from incoherent ones. They found that coherent dispersoids were toward an equilibrium size at each temperature tested, and incoherent dispersoids are destroyed at low temperature but survived while shrinking in size at higher temperatures. In addition, the coarsening of nano-oxides was accompanied with a loss of the coherent atomic structure of the oxide/matrix interface [12].

Therefore, all of these studies show that there are difficulties in retaining nano-oxide dispersion under irradiation at a nanoscale. This might lead the ODS steels to exhibit a possible loss of nanostructuration in service conditions. This paper proposes to detail the behaviour of the nano-oxides when the material is subjected to both thermal annealing and irradiation. A theoretical background is first proposed to introduce the enhanced and inverse Ostwald ripening mechanism.

## 2. Materials and Methods

All the results presented in this paper were obtained on ferritic ODS steels developed at CEA and elaborated by mechanical alloying. This process consisted of the milling under a hydrogen atmosphere of both pre-alloyed Fe-Cr-W-Ti powder and Y_2_O_3_ powder in an attritor. After powder milling, the alloy was hot extruded at 1100 °C and then annealed at 1050 °C for 1 h. The chemical compositions of the two studied materials are presented in Table 1. Mn, Ni and Si elements were in minor concentrations that were initially present in the Fe-Cr-W-Ti powder, while C resulted from contamination. Those two alloys are called Fe-14Cr ODS and Fe-18Cr ODS hereafter. 

TEM observations were conducted on a JEOL-2100 microscope (JEOL LTD, Tokyo, Japan) operating at 200 kV, while HRTEM was performed on both JEOL-2010F and CS-corrected (probe and image) JEOL-neoARM microscopes operating at 200 kV. Chemical analysis was performed using the neoARM microscope equipped with double-Centurio EDS (Energy Dispersion Spectroscopy) detectors (JEOL LTD, Tokyo, Japan). TEM disks were punched from 60 µm-thick, mechanically polished foils and electropolished using a 10% perchloric acid, 90% ethanol solution at −10 °C in a Struers Tenupol thinning device. The Focused Ion Beam (FIB) technique was also used to extract a standard cross-section preparation from a virgin ODS sample with an FEI Helios SEM/FIB dual beam microscope (Thermo Fisher Scientific, Waltham, MA, USA).

Atom Probe Tomography (APT) analysis was performed on a CAMECA LEAP 4000X HR (Ametek, Inc., Berwyn, PA, USA) in laser pulsing mode; a base temperature of 50 K, laser pulse energy of 80 pJ and pulse repetition rate of 250 kHz were used during the experiments.

The ion irradiations were conducted at the CEA-JANNuS Saclay facility (CEA, Saclay, France) [13] using 500 keV Fe^+^ self-ions with a flux of 2.6–2.8 × 10^12^ ions·cm^−2^s^−1^. The specimens had a thin-foil geometry; were maintained at nominal temperatures of 300 °C, 400 °C and 500 °C; and were tilted by 15° with respect to the incoming ion beamline. The depth profile of irradiation damage using 500 keV Fe^+^ ions can be found in [7]. The mean doses reached within the first 100 nm were estimated to be roughly 75 dpa [7] and 150 dpa [7] for a fluence of 4.4 × 10^16^ ions·cm^−2^ and 8.9 × 10^16^ ions·cm^−2^, respectively.

**Figure 1:**
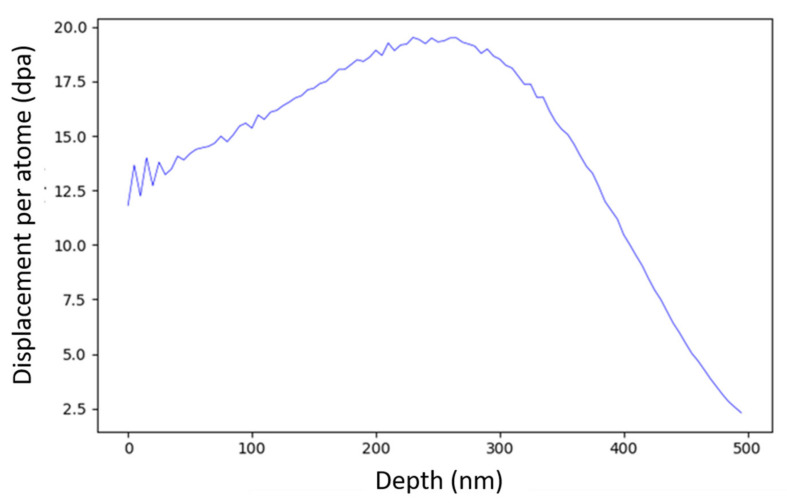
Depth profile of irradiation damages (displacement per atom) in Fe calculated by Iradina [14] using 4000 keV-Au^2+^ ions with a fluence of 4.0 × 10^15^ ions·cm^−2^.

In situ ion irradiations were conducted at the JANNuS-Orsay facility (Université Paris-Saclay, Orsay, France) [13] at IJClab equipped with a FEI Tecnai G^2^ 20 TEM (Thermo Fisher Scientific, Waltham, MA, USA) operating at 200 kV and coupled with the ARAMIS ion accelerator. Irradiations were performed at room temperature and at 500 °C. At room temperature, the irradiations were conducted using 4000 keV Au^2+^ ions with a flux of 2.0 × 10^11^ ions·cm^−2^s^−1^. The depth profile of the irradiation damage was calculated using Kinchin–Pease mode from the Iradina software [14] with 40 eV displacement energy. The irradiation damage profile is presented in Figure 1. At 500 °C, the irradiations were conducted using 150 keV Fe^+^ ions with a flux of 2.9 × 10^12^ ions·cm^−2^·s^−1^. The corresponding irradiation profile is presented elsewhere [15].

## 3. Results

### 3.1. Theoretical Background: The Irradiation Modified Gibbs–Thomson Relation

Before considering the results, a theoretical background is necessary to introduce the effect of ion bombardment on nanoparticle dispersion. After nucleation and growth, nano-precipitates start to coarsen. This late stage of the evolution of the isolated nano-precipitates is called Ostwald ripening and consists of the shrinking of small nano-precipitates to the benefit of the largest ones. This process is driven by the minimization of the surface energy associated with the precipitate/matrix interface [16]. In the second phase, coarsening rates are controlled by both kinetics and the solubility in the neighbouring matrix of the rate-controlling solute element [17]. The interface curvature of a precipitate with radius *R* modifies the solubility limit given by the phase diagram: this is the so-called Gibbs–Thomson effect. The corrected solubility is then given as a function of *R* [18]:

(1) $$C\left(r\right)=C_{\infty }exp\left(2\gamma V_{m}/RR_{G}T\right)$$

where $C_{\infty }=C_{0}exp\left(-E_{s}/kT\right)$ is the equilibrium solubility with $E_{s}$ as the activation energy, γ is the interface energy, $V_{m}$ is the molar volume and R is the precipitate radius, while $R_{G}$ and T are the gas constant and absolute temperature, respectively. For small values of the capillary length $R_{C}=2\gamma V_{m}/R_{G}T$, the solubility can be linearly approximated as

(2) $$C\left(r\right)=C_{\infty }\left(1+R_{C}/R\right)$$

Therefore, due to the interface curvature, the solubility increases with decreasing R, and coarsening occurs in the system of disperse precipitates by the flow of solutes from smaller dissolving precipitates to larger growing precipitates [17].

The mathematical treatment of the equation describing the coarsening of particles was carried out simultaneously and independently by Lifshitz and Slyosov [19] and Wagner [20]. When the diffusion in the matrix is the rate-controlling step, they obtained the same results, which were as follows [21]:

- A quasi-stationary particle size distribution is approached asymptotically;
- After the quasi-stationary condition is reached, the third power of the mean particle size increases linearly with time, with a unique slope, according to the following equation:

(3) $${\bar{R}}^{3}\left(t\right)-{\bar{R}}^{3}\left(0\right)=K_{th}\left(T\right)t=\frac{8\gamma D^{th}C_{\infty }V_{m}^{2}}{9R_{G}T}t\;$$

where γ, $C_{\infty }$, $V_{m}$, $R_{G}$ and T have been previously defined, while t is the time and $D^{th}=D_{0}exp\left(-E_{m}/kT\right)$ is the diffusion coefficient of the rate-controlling solute.

Under irradiation, the coarsening of the precipitates is significantly modified owing to the collision cascade that knocks out and ejects precipitate atoms from their original position. For atoms close to the interface, they will be forced to leave the precipitates and will be deposited into the surrounding matrix. Therefore, the detachment of atoms from nano-precipitates will be both thermally activated with respect to the interface energy barrier and caused by the displacement of the collision cascade, independently of the interface energy barrier. Consequently, the solute solubility under irradiation is not solely ruled by the interface curvature but also by the displacement cascade, which then must be modified.

Heinig et al. [22] found that with irradiation at a damage rate qϕ, the solute concentration around a nano-precipitate obeys a similar law in the form of the Gibbs–Thomson relation, where only the solubility $C_{\infty }^{I}$ and capillary length $R_{c}^{I}$ are modified; this new relation is expressed as

(4) $$C^{I}\left(R\right)=C_{\infty }^{I}\left(1+R_{C}^{I}/R_{0}\right)\;$$

**Figure 2:**
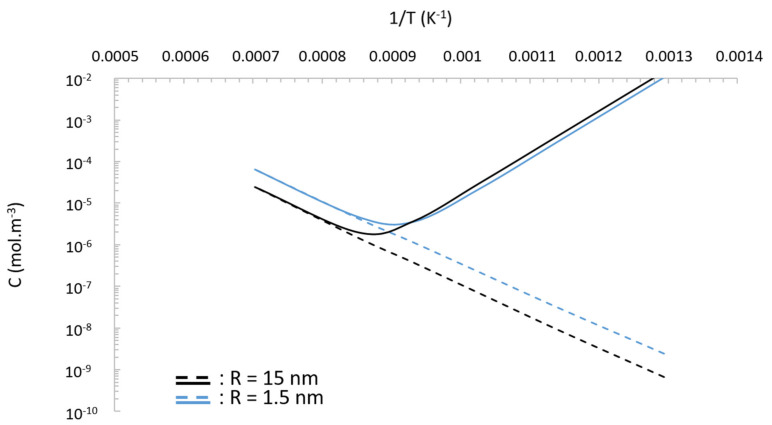
Dependence on the irradiation temperature irradiation of the yttrium solute concentration produced by recoil resolution and by thermal solubility for pyrochlore-type precipitate.

With

$$C_{\infty }^{I}=C_{\infty }\left(1+\Delta \right)$$

$$R_{C}^{I}=\left(R_{C}-5\lambda \Delta /4\right)/\left(1+\Delta \right)$$

$$\Delta =q\phi \lambda^{2}/D^{th}C_{\infty }$$

where qϕ and λ are the damage rate and the mean displacement of ejected atoms, respectively. Thus, according to this irradiation-modified Gibbs–Thomson relation, the nano-precipitate evolution under irradiation is expected to be completely different from equilibrium thermodynamics. Heinig et al. [22] found that the most striking feature of Equation (4) is the change of capillary length, which can become negative with a high damage rate qϕ, large mean displacement λ or low irradiation temperature. Figure 2 shows the behaviour of a pyrochlore-type precipitate embedded within a ferritic matrix, where the dependence of $C^{I}\left(R\right)$ on T has been plot for two precipitates with radii of 1.5 nm and 15 nm and at a damage rate of 6.5 × 10^−3^ dpa·s^−1^. The dashed line is the usual solubility for a curved interface—i.e., the conventional Gibbs–Thomson relation, $C\left(R\right)$—whereas the thick solid curve is the plot of the solute concentration at the precipitate interface under irradiation, $C^{I}\left(R\right)$. The coefficient values used to calculate the solubility are summarized in Table 2. Yttrium is considered as the rate-controlling element owing to its low diffusivity.

As shown by Heinig et al. [22], two distinguished behaviours can be observed. With increasing temperature, the parameter Δ approaches zero exponentially; i.e., at high temperature, the behaviour is ruled by the conventional Gibbs–Thomson relation. In this regime, nano-precipitates have a positive capillary length, and conventional Ostwald ripening is expected where large precipitates grow at the expense of small ones. However, for a critical temperature, $T_{C}$, the capillary length becomes negative, leading the small precipitates to grow at the expense of the large ones. This regime is called inverse Ostwald ripening.

Thus, under irradiation, a dispersion of pyrochlore-type precipitates could behave differently with respect to the irradiation conditions. The modification by the collision cascade of the solute concentration at the precipitate/matrix interface could drive the coarsening process to alternate between conventional and inverse Ostwald ripening.

It should be borne in mind that this model does not propose an exhaustive view of the irradiation response of nano-precipitates, as, for instance, the change of diffusivity of elements along with the irradiation are not taken into account. However, the model proposes a correct and simple illustration of the behaviour of the nano-precipitate under irradiation.

### 3.2. Characterization of the Nano-Oxide Dispersion

**Figure 3:**
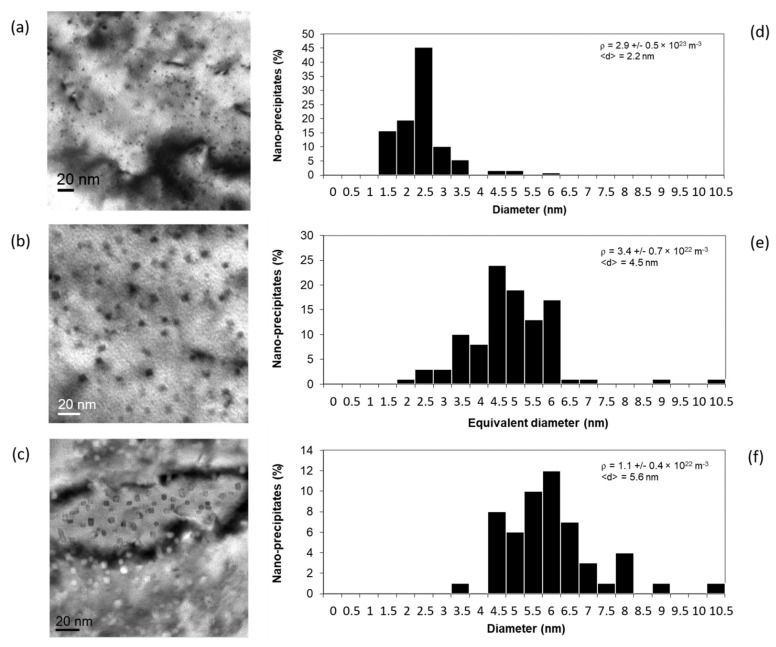
Bright field TEM image with its corresponding histogram: (**a**,**d**) as-received material, (**b**,**e**) after thermal annealing (1300 °C, 1 h), (**c**,**f**) after irradiation up to 150 dpa at 500 °C.

Figure 3a is a bright-field TEM image showing the nano-oxide distribution in the as-received Fe-14Cr ODS steel. One can observe that the nano-precipitates are homogeneously dispersed within the ferritic matrix. Their size ranges from 1 nm to 6.5 nm for an average diameter of 2.2 nm, as depicted by the histogram in Figure 3d, while the nano-oxide density is estimated at 2.9 ± 0.5 × 10^23^ m^−3^. The nano-oxides appear rather spherical, even though their shape remains difficult to appreciate considering their small size.

**Figure 4:**
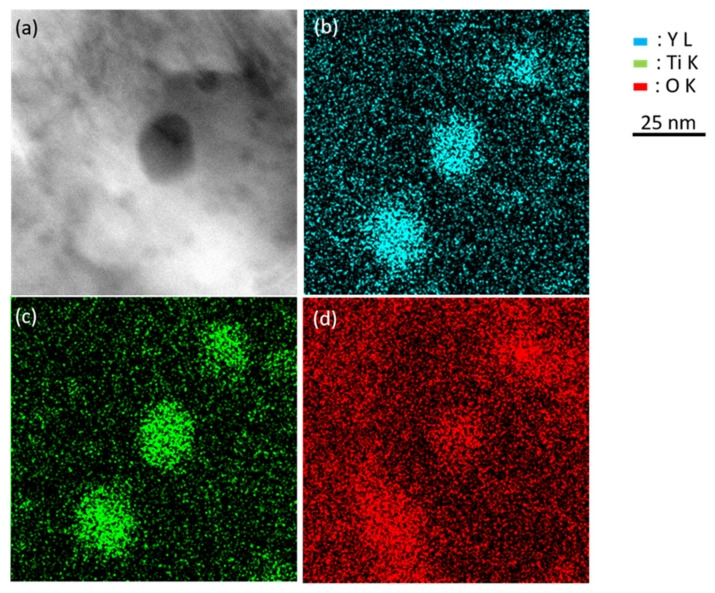
Chemical map of the nano-oxides from EDS acquisition: (**a**) ADF STEM image, (**b**) Y L ac-quisition, (**c**) Ti K acquisition, and (**d**) O K acquisition.

The chemical map (Figure 4), obtained in the as-received Fe-18Cr ODS after the EDS acquisition of the rays generated by emission from the energy-level shells Y L, O K and Ti K, shows that the nano-oxides are Y, Ti and O-rich particles (those in the Fe-18Cr ODS oxide are slightly larger than in Fe-14Cr ODS). 

**Figure 5:**
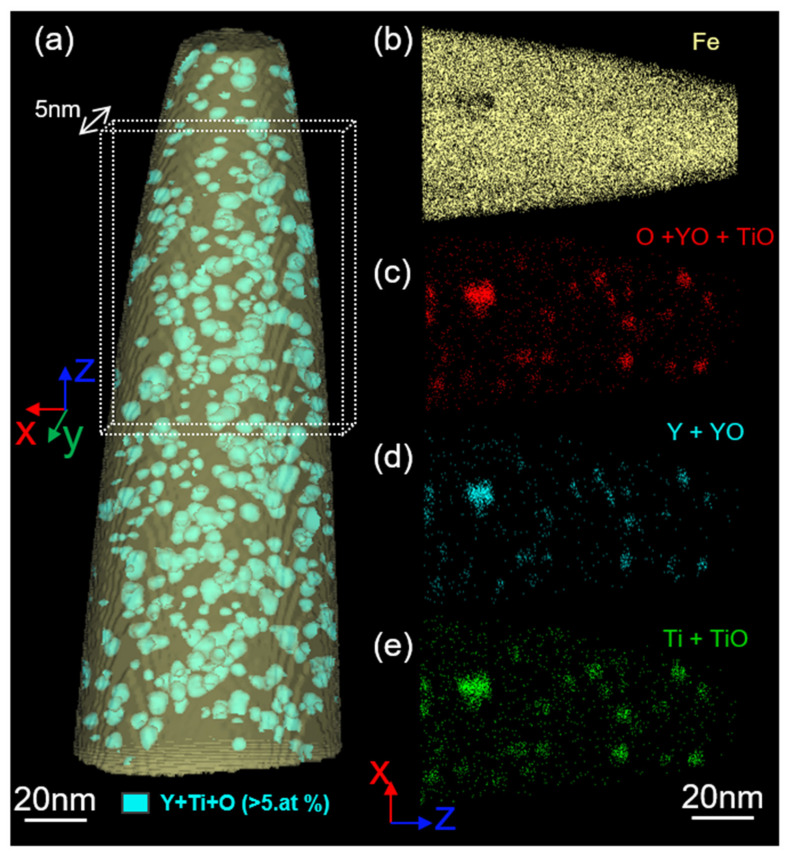
Three-dimensional APT reconstruction of as-received Fe-14Cr ODS: (**a**) an iso-composition surface >5 at.% of O+Y+Ti in blue was used to highlight nano-oxides, (**b**–**e**) zoomed in insert of region of interest showing map of Fe, O, Y and Ti atoms in as-received Fe-14Cr ODS steel.

A representative APT reconstruction of the as-received Fe-14Cr ODS steel is summarized in Figure 5. The nano-oxide dispersion in the APT-reconstructed volume in Figure 5a is highlighted by an iso-composition surface (in blue) delineating the regions containing more than 5 at% of Ti + Y + O. Figure 5b–e shows a close-up of the reconstruction (slice along XZ of 5 nm thickness) taken from the region of interest (ROI) highlighted by the dashed line square in Figure 5a. From these maps, nanoclusters rich in Y, Ti and O with different sizes are clearly visible compatible with TEM observation. The Y:Ti ratio in the nano-oxide is around 0.5 regardless of the size, and no phase other than (Y, Ti, O)-enriched particles seems to be present. The nano-oxide density estimated by APT is found to be 4.7 ± 0.5 × 10^23^ m^−3^, while the average diameter is found to be 2.0 nm. Those values are in good accordance with both the density and average diameter measured by TEM. 

**Figure 6:**
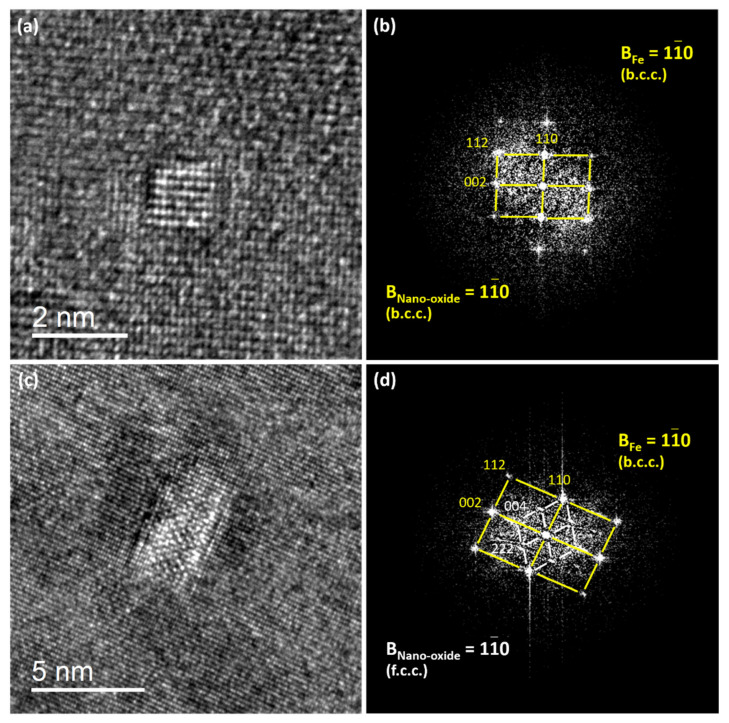
HRTEM images of both nano-cluster (<1.5 nm) and nano-oxide embedded within the ferritic matrix: (**a**) HRTEM image of a nano-cluster, (**b**) corresponding FFT, (**c**) HRTEM image of a nano-oxide, (**d**) corresponding FFT.

**Figure 7:**
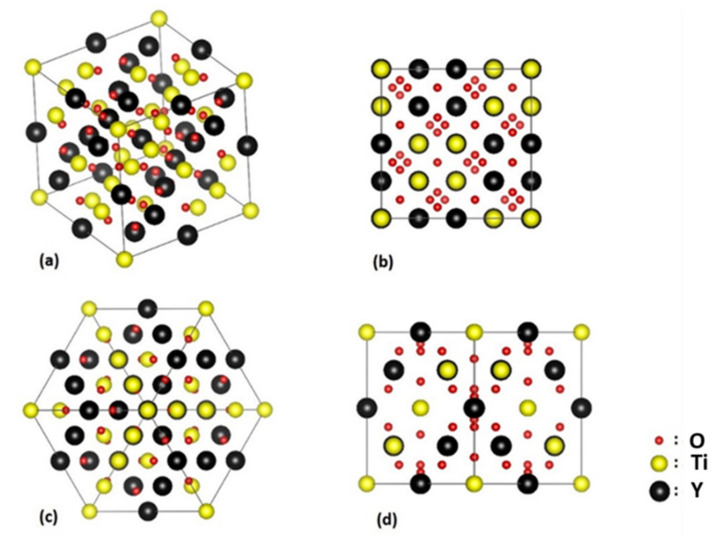
Pyrochlore-type lattice structure: (**a**) general view, (**b**) observed along the (001) direction, (**c**) observed along the (111) direction, (**d**) observed along the (011) direction.

The lattice structures of very small nano-oxides (<1.5 nm), called nano-clusters [26], are difficult to image by HRTEM owing to their very small size. However, Figure 6a proposes a HRTEM image of a 1.3 nm-sized nano-cluster, where atomic planes of both the cluster and matrix are resolved. The matrix is orientated along the $\langle 1\bar{1}0\rangle$ direction. In the Fast Fourier Transform (FFT) (Figure 6b) of the HRTEM image, no additional spots from the nano-cluster are distinguishable since the lattice structure and the interplanar distance are similar to those of the matrix. This result suggests that at this very small size, the nanocluster adopts the α-Fe bcc lattice structure imposed by the matrix, as already proposed in [26]. Further, one may observe in Figure 6a that the nanocluster is fully coherent with the matrix, as suggested in [26]. However, after slight growth, the nanocluster becomes a nano-oxide, displaying its own structure (Figure 6c), which corresponds to the equilibrium pyrochlore-like phase [25,26]. The lattice parameter of the pyrochlore phase is 1.01 nm [25], while the space group is Fd$\bar{3}$m. The lattice structure of a Y_2_Ti_2_O_7_ pyrochlore oxide is closely related to the fluorite structure, except that there are two cation sites and one-eighth of the anions are absent (8a). The Y and Ti metal cations occupy the 16d(1/2, 1/2, 1/2) and 16(0, 0, 0) sites, respectively, while the oxygen atoms are in the 48f(x, 1/8, 1/8) and 8b(3/8, 3/8, 3/8) positions [25]. Figure 7 illustrates the atomic arrangement of the pyrochlore-type lattice structure. Figure 6c shows an HRTEM image of a nano-oxide embedded in a matrix oriented along the $\langle 1\bar{1}0\rangle$ direction. In the FFT (Figure 6d), the additional spot corresponds to the atomic planes of the oxide. The zone axis corresponding to the precipitates displays 〈110〉 fcc symmetry, while the two measured interplanar distances are found to be 0.254 nm and 0.304 nm. These values are in good accordance with the 〈400〉 (0.25 nm) and the $\langle \bar{2}\bar{2}2\rangle$ (0.29 nm) pyrochlore distances [26]. The nano-oxide displays a cube-on-cube relationship with the matrix, as expected owing to the full coherency of the nanocluster parent phase (Figure 6a).

### 3.3. Evolution of the Nano-Oxides Distribution during Thermal Annealing

Figure 3b is a bright-field TEM image showing the nano-oxides dispersion after thermal annealing at 1300 °C for 1 h. It can be clearly observed that particles have grown since their size is now ranging from 2 nm to 10.5 nm with an average diameter of 4.5 nm (Figure 3e). Since the shape of the particle is no longer spherical but rather cubical, an equivalent diameter $\left(d_{eq}=4a/\sqrt{\pi }\right)$ was considered, with 2a being the edge length of the cubical particle. Further, the density significantly decreased from 2.9 ± 0.5 × 10^23^ m^−3^ to 3.4 ± 0.7 × 10^22^ m^−3^. To determine the extent to which the coarsening regime corresponds to this increase in size, we first need to calculate the interfacial energy of the nano-oxides. 

#### 3.3.1. Interface Energy Calculation Deduced from Morphological Transition

**Figure 8:**
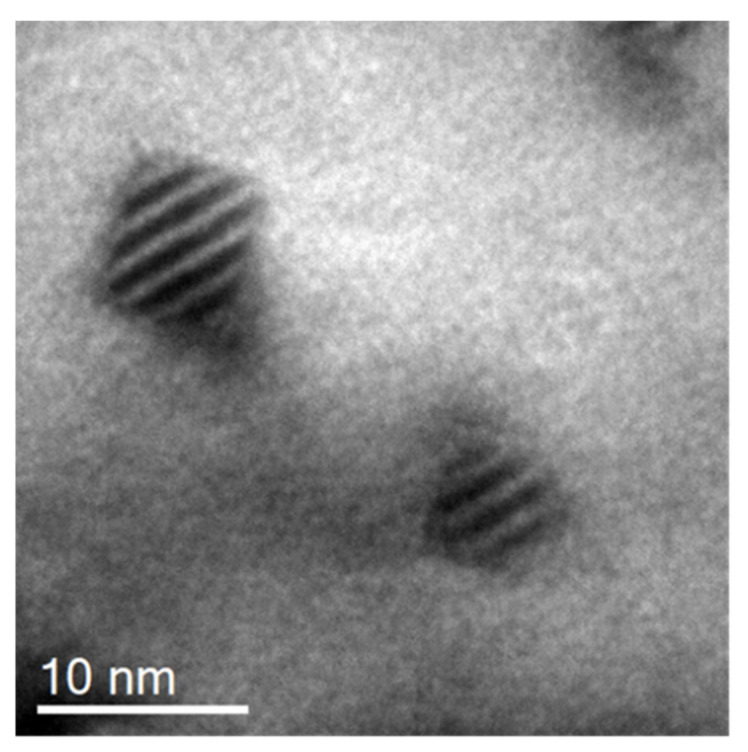
TEM image of two cubical pyrochlore oxides after coarsening at 1300 °C.

As previously mentioned, during coarsening at 1300 °C, the particle shape has evolved from spherical to cubical. Figure 8 shows two cubic oxide particles at high magnification with a near-perfect cubical shape, suggesting that they have almost completed their transition. The origin of this shape bifurcation lies in the misfitting coherent relationships of the small nano-oxides embedded within the ferritic matrix. When the oxide is small, this energy can be neglected; however, with nano-oxide growth, the elasticity energy at the precipitate/matrix interface predominates over the interfacial energy and drives the particle to adopt a shape in common symmetry with the matrix. This shape transition allows the accommodation of the strain energy. Since they initially display a cube-on-cube orientation relationship with the matrix [25], the nano-oxides adopt a preferred cubic shape and introduce dislocations at the interface to relieve the misfit strain. The nano-oxides then become semi-coherent [25].

Thanks to the elastically driven morphology, we find that the interface energy of the nano-oxides can be estimated by considering the work of Khachaturyan et al. [27].

For ease of comparison with a cube, Khachaturyan [27] considered the volume of a sphere equal to ${\left(2a\right)}^{3}$ while the surface area becomes $S=4a^{2}{\left(36\pi \right)}^{1/3}$.

According to Khachaturyan et al. [27], the total energy of spherical particles with volume ${\left(2a\right)}^{3}$ is expressed as

(5) $$\Delta F\left(sphere\right)=0.709E_{1}{\left(2a\right)}^{3}+\gamma {\left(36\pi \right)}^{1/3}{\left(2a\right)}^{2}$$

While the total energy of a cubic particle of the same volume is expressed as [27]

(6) $$\Delta F\left(cube\right)=0.558E_{1}{\left(2a\right)}^{3}+6\gamma {\left(2a\right)}^{2}$$

In both $\Delta F\left(sphere\right)\;$and $\Delta F\left(cube\right)$, the first term corresponds to the elastic energy while the second term is relative to the interfacial energy, $E_{1}$ is a function of the elastic constants and the misfit strain [25], and γ is the interfacial energy. 

It follows that the sphere becomes metastable and transform into a cube when

$$\Delta F\left(sphere\right)\geq \Delta F\left(cube\right)$$

Which corresponds to

(7) $$2a\geq 7.7\left(\gamma /E_{1}\right)$$

The critical radius for which the nano-oxide displays a full cubical shape is estimated to be roughly 1 nm [25]. When considering the (110)Fe and (110)Y_2_Ti_2_O_7_ mismatching plane, the resulting misfit strain is found to be 12.6% [25]. Therefore, by replacing those values in Equation (7) and by replacing the value of $E_{1}$ given elsewhere [25], we find that the interfacial energy of the nano-oxide embedded with cube-on-cube orientation is 290 mJ·m^−2^. By using a more accurate approach [25], the interface energy has been refined to a value of 260 mJ.m^−2^.

#### 3.3.2. Ostwald Ripening Process

After annealing at 1300 °C, the change in particle interspacing in Figure 3b suggests the consumption of particles by the Ostwald ripening process. When following this coarsening process, the interface mobility-controlled kinetics of the growing particles are described by the Lifshitz–Slyozov–Wagner (LSW) [19,20] theory, expressed in the form described above in Equation (3). However, to confirm that the coarsening particles’ kinetics scale as$\;t^{1/3}$, we first consider a coarsening expression with the general form [17]

(8) $$R^{p}\left(t\right)-R_{0}^{p}\left(t\right)=K_{th}\left(T\right)t$$

where $K_{th}\left(T\right)$ is the rate coefficient containing a combination of materials properties as described in Equation (3). The experimental data of the radius evolution with different aging temperatures and times can be found elsewhere [28,29] (both of these authors studied similar CEA ODS steels with identical initial average radii of nano-oxides: 1.4 nm for [28] and 1.3 nm for [29]). These values, presented in Table 3, were obtained by means of SANS (Small Angular Neutron Scattering) on a Fe-14cr ODS. A comparison between TEM and SANS radius values is also presented in Table 3 for two conditions, in order to prove that both techniques result in similar radius values within acceptable errors [28].

**Figure 9:**
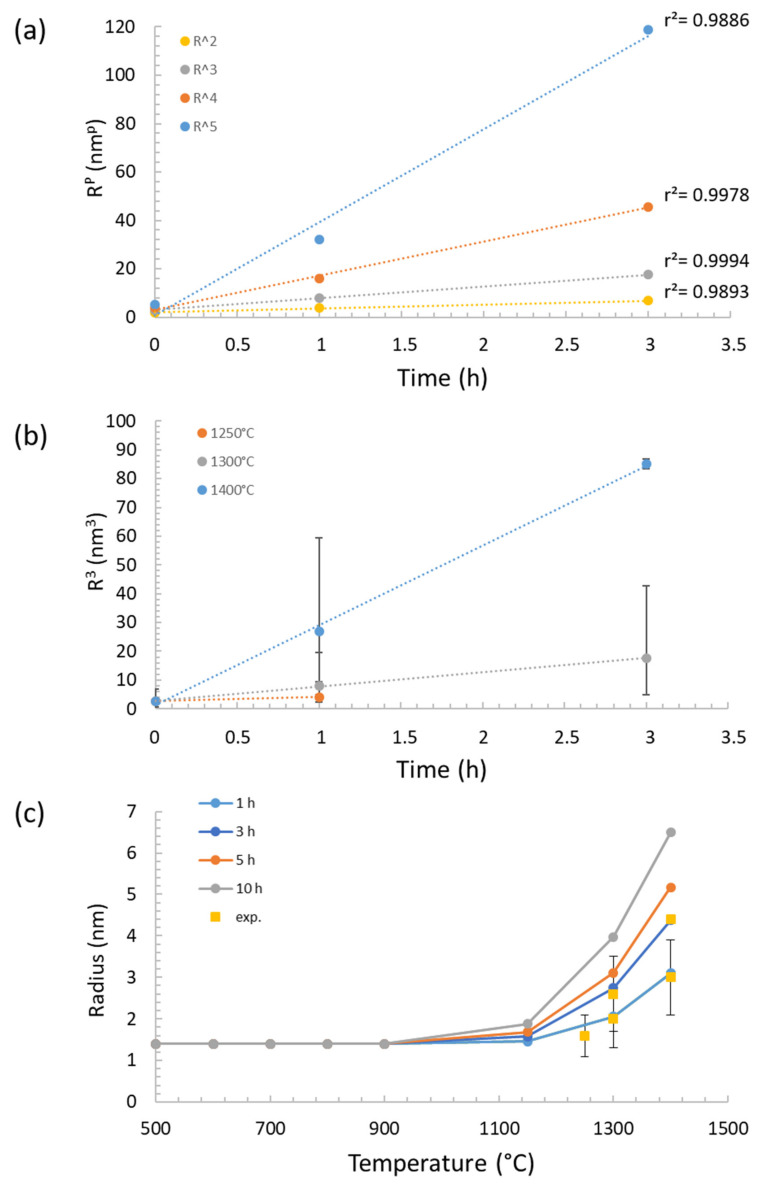
Ostwald ripening of the nano-oxides: (**a**) evolution of the radius elevated at the power *p* (*p* = 2, 3, 4, 5) against time for a thermal annealing at 1300 °C; (**b**) evolution of the cube radius against time for thermal annealing at 1250 °C, 1300 °C and 1400 °C; (**c**) plot of the calculated and experimental radii against temperature for various annealing durations.

Figure 9a presents the plot of the nano-oxide radius elevated at the power p with an aging time at 1300 °C. The value p ranges from 2 to 5, and for each plot, the coefficient of determination *r^2^* is calculated. The better the linear regression fits the data, the closer the value of *r^2^* is to 1. We found that *r^2^* approaches 1 for the value p = 3. However, all the plots presented in this graph, with p ranging from 2 to 5, can be considered to have a linear slope, since all values of *r^2^* are very close to 1. Further, the plots rely on only three points, while more experimental values would have been required to correctly establish the value of p. Therefore, no clear conclusion is possible from this approach. In addition, since the density values are not available [28], the linear evolution of the density with the inverse of the annealing time cannot be assessed. However, since the value of *r^2^* is closer to 1 for p = 3, it is considered in the following as a first approximation that the radius evolution scales as $t^{1/3}$ as expected by the LSW theory. Thus, Ostwald ripening is supposed to be the coarsening process of the nano-oxides. Figure 9b shows the evolution of the cube radius against time for 1250 °C, 1300 °C and 1400 °C aging temperatures. The linear evolution is also confirmed for coarsening at 1400 °C.

By replacing the values from Table 2 in the LSW equation, the values of the rate coefficient $K_{th}\left(T\right)\;$for p = 3 are given in Table 4.

By considering these values of $K_{th}\left(T\right)$, Figure 9c presents a simulation of the expected radius at aging times ranging from 1 h to 10 h. Figure 9c also presents a comparison between experimental values of radii with those calculated from the LSW theory. We can conclude that experimental and calculated values match very well for both 1 h and 3 h aging times at the various temperatures.

Since the values from Table 2 reproduce the radius growth during coarsening well, one may conclude that a good estimation of the different parameters of Equation (3) has been achieved, especially the interfacial energy that we deduced from the elasticity-driven morphology phenomenon. However, a classical theory is used to illustrate the increase in size of the nano-oxides. Although this reproduces the radius evolution quite well, one may note that in the classic theory, the interfacial reaction kinetic is ruled by the interface energy. The higher the interface energy is the easier atom can precipitate at the particle interface, regardless of the atom element and of the type of vacant site in which it condensated. However, in the case of oxide, stoichiometric composition has to be respected. Therefore, the condensation of Y and Ti atoms becomes also a limiting factor for the growth kinetics owing to the oxide stoichiometry that has to be respected: a Ti atom can only condensate at a Ti vacant site and a Y atom can only condensate at a Y vacant site to respect the Y_2_Ti_2_O_7_ atom arrangement.

This phenomenon can sluggish the kinetic even though the interfacial energy is high. The classical theory does not take this into account; thus a more complex model should be applied to consider this phenomenon correctly. In addition, the nano-oxides are not initially stoichiometric oxides, as the APT results showed (the ratio Y:Ti is found to be 0.5 instead of 1); this particularity should also affect the growth kinetics. 

In conclusion, Ostwald ripening has been identified as the coarsening mechanism, but more data are recommended to validate this. However, one may note the remarkable coarsening resistance of the nano-oxides, since Figure 9c shows that precipitates start to increase in size only above 1100 °C. Both low Y diffusion and low interfacial energy combined with the complexity of the Y_2_Ti_2_O_7_ lattice structure are certainly at the root of the sluggish coarsening kinetics.

### 3.4. Evolution of the Nano-Oxide Distribution under Irradiation

Since thermal aging requires a temperature high enough to trigger the coarsening of the nano-oxides, a question arises concerning the behaviour under irradiation of such thermal-resistant particles. Depending on the irradiation temperature, several behaviours emerge and are described below.

#### 3.4.1. Irradiation Response of the Nano-Oxides at Low Temperature

1. Radiation-induced dissolution

As described in Equation (4), under irradiation, the interface curvature is no longer solely responsible for the increase in solute concentration at the interface, but recoil resolution largely contributes to it. At low temperature, this phenomenon is accompanied with the dissolution of the particle, since relocated atoms are frozen and remain in the matrix [3]. To prove this phenomenon, a single particle in a Fe-18Cr ODS was followed with TEM during in situ Au^2+^ ion irradiation. This irradiation was conducted at room temperature where the ballistic effect could be isolated. After 5.6 h, the irradiation damage reached an average dose of 15 dpa within the first 100 nm, as depicted in Figure 1. 

**Figure 10:**
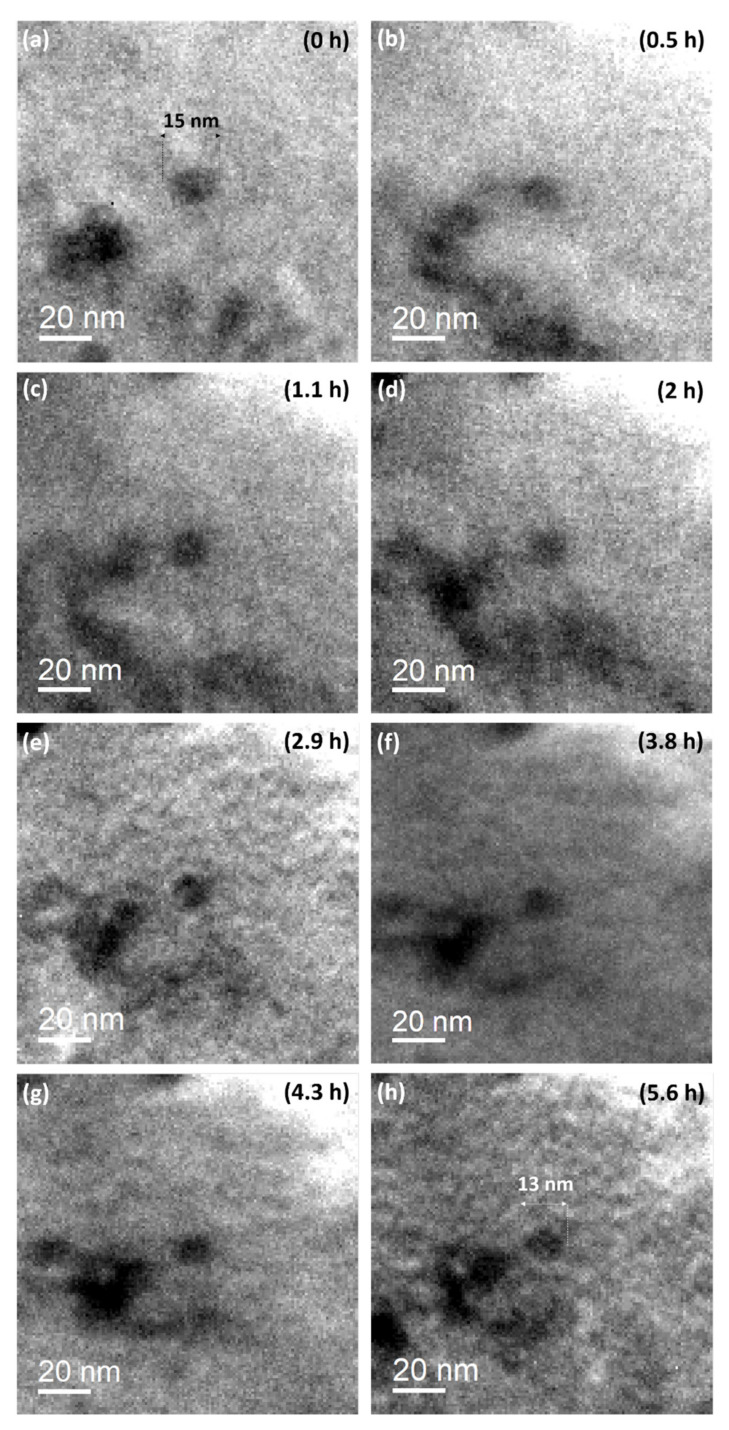
In situ observation of the evolution of a nano-oxide during 4 MeV Au^2+^ irradiation: (**a**) before irradiation, (**b**) after 0.5 h (1.3 dpa), (**c**) after 1.1 h (3 dpa), (**d**) after 2 h (5 dpa), (**e**) after 2.9 h (8 dpa), (**f**) after 3.8 h (10 dpa), (**g**) after 4.3 h (12 dpa), and (**h**) after 5.6 h (15 dpa).

Figure 10a shows the nano-oxide prior to irradiation while Figure 10b–h show the oxide under irradiation at various duration. The initial diameter is estimated at 14.4 nm. After 2.9 h—i.e., almost 8 dpa—the nano-oxide appears smaller (Figure 10e) and is measured at 13 nm, while after 4.3 h—i.e., almost 12 dpa—the oxide is measured at 10.9 nm (Figure 10g). Finally, with a correct and stabilized image condition, Figure 10h reveals that the oxide has shrunk to 12.7 nm during the 5.6 h of irradiation. 

**Figure 11:**
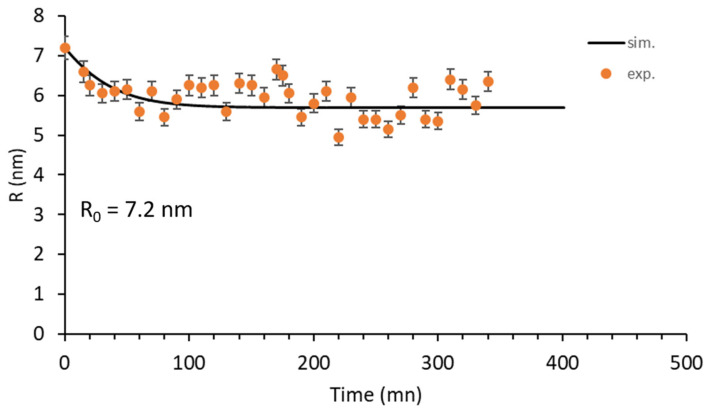
Experimental and theoretical dissolution rate of the nano-oxide presented in Figure 9 during in situ 4 MeV Au^2+^ ion irradiation.

Figure 11 shows the continuous dissolution of the nano-oxide along with the irradiation. The error between the actual and measured value was estimated at 4% for a LaB_6_ electron gun [30]. This takes into account the lack in definition of the particle perimeter caused by the imprecise focus setting during irradiation acquisition. The plot shows that the majority of the solute displaced atoms are ejected from the oxide at the beginning of the irradiation, during the first hour. After this, the particle slowly continues to dissolve until it reaches the steady state. However, the behaviour of more than 12 particles with radii ranging from 4 to 40 nm were followed during this in situ irradiation experiment. The results are not presented in this paper, but all the particles presented dissolution. Further, particles with same initial size presented comparable kinetics after 5.6 h of irradiation. Therefore, the oxide behaviour presented in Figure 11 can be considered as reliable, and the initial and final size of the oxide are correctly measured under stabilized conditions; however, the kinetics are imprecise owing to the difficulty to obtain correct conditions for image acquisition during irradiation.

The theoretical model used to reproduce the dissolution rate is the model proposed by Frost and Russel [31] and recently modified by Vu et al. [32] by replacing the constant damage generation rate by a variable rate taking into account the shrinking of the particle with irradiation. Therefore, the dissolution rate is expressed as [32]

(9) $$\frac{dR}{dt}=\frac{1}{R}\frac{L}{L-R}\frac{\lambda_{m}^{2}S_{0}}{48}\left[\lambda_{m}\left(\frac{1}{R}-\frac{1}{R_{m}}\right)+\left(\frac{\lambda_{m}}{R_{m}}-4\right)exp\left(-BR_{m}\right)-\left(\frac{\lambda_{m}}{R}-4\right)exp\left(-BR\right)\right]$$

where R is the particle radius, 2L is the interparticle spacing, $\lambda_{m}$ is the largest recoil distance of the ejected atom, $S_{0}$ is the recoil generation rate, $R_{m}$ stands for the steady state particle radius and B is a constant.

The main limitation of Equation (9) lies in the fact that many parameters are needed to model the time evolution of the mean radius of particles. The solution of Equation (9) was obtained from a Runge Kutta (RK) integration (four-order RK method) with $2R_{0}$ as the initial condition. Values of the fitted parameters are summarized in Table 5, while the corresponding plot appears in a solid line in Figure 11. The experimental points fluctuate as a result of the image acquisition conditions, which limits a precise fit of the curve. However, the fit shows good agreement with the experimental values and reproduces the general dissolution trend well.

From this in situ ion irradiation experiment, we learned that the recoil atoms ejected from the oxide are not only responsible for the oxide dissolution at low temperature but, consequently, are also responsible for the increase in solute concentration at the interface, as described in Figure 2. At higher temperature, these recoil atoms are free to migrate in the matrix. Owing to radiation-enhanced diffusion [3], and depending on the driving force to which they are subjected, they will move back to the original oxide or they will move toward other particles where they will condensate. If the second process dominates, coarsening by enhanced or inverse Ostwald ripening, as described above, will then be triggered.

#### 3.4.2. Irradiation Response of the Nano-Oxides at High Temperature

1. Radiation-induced inverse Ostwald ripening

**Figure 12:**
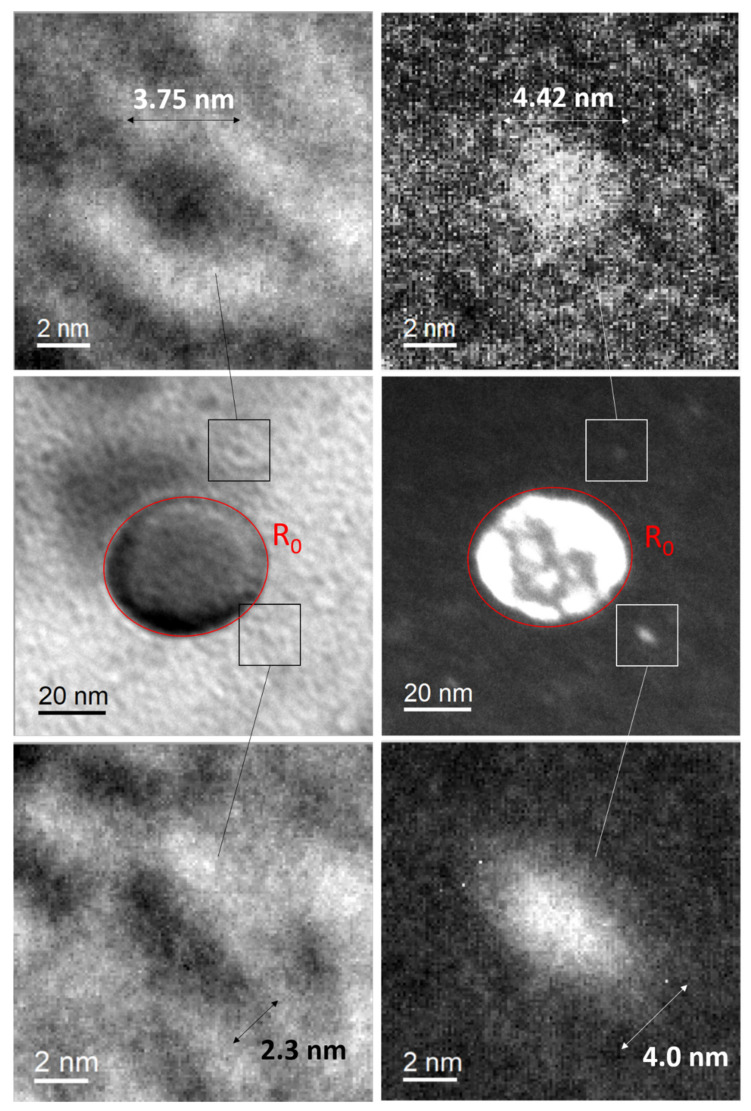
Radiation-induced inverse Ostwald ripening after 150 keV Fe^+^ ion irradiation: (**a**) bright-field image of a large oxide surrounded by nano-oxides before irradiation, (**b**) dark-field image of the same particles after irradiation (4 dpa), (**c**) a neighbouring nano-oxide before irradiation, (**d**) the same neighbouring nano-oxide after irradiation (4 dpa), which appears larger, (**e**) another neighbouring nano-oxide before irradiation, (**f**) the same nano-oxide after irradiation (4 dpa), which appears larger.

The TEM images presented in Figure 12 were acquired during an in situ irradiation experiment conducted at 500 °C using Fe^+^ ions; the damage profile is described elsewhere [15]. Figure 12a shows a large oxide, with an initial diameter estimated at 48.5 nm. Figure 12b is the same oxide after irradiation up to 4 dpa, but it has shrunk as its diameter is now estimated at 46.3 nm. To illustrate this change in diameter, the initial diameter of the oxide is reported as a red circle in Figure 12b. As we previously observed, this dissolution is probably accompanied by an increase in solute concentration around the oxide that may diffuse toward surrounding particles and trigger their growth. Two nano-oxides are in the close vicinity of the oxide, as shown in Figure 12a and presented in high magnification in Figure 12c,e. Before irradiation, the characteristic length of the nano-oxide presented in Figure 12c is 2.3 nm, while the characteristic length of the other nano-oxide presented in Figure 12e is 3.75 nm. After irradiation, the TEM images suggest that both of these nano-oxides have grown. Figure 12d shows that the nano-oxide size has evolved from 2.3 nm to 4 nm, while Figure 12f shows that the other nano-oxide has evolved from 3.75 nm to 4.42 nm. Therefore, the shrinking of the large oxide combined with the growth of two neighbouring nano-oxides can be interpreted as an inverse Ostwald ripening, even if there is no clear evidence that the growth of the two neighbouring nano-oxides is due to the condensation of atoms ejected from the large oxides. Further, in Figure 12a, the nano-oxide contrast is poor but appears better in the dark-field contrast of Figure 12b, which limits the correct estimation of the initial size of the nano-oxides.

2. Radiation-enhanced Ostwald ripening

We observed that inverse Ostwald ripening arose in the particular configuration where a large oxide is surrounded by smaller ones. In the following, we focus on the small sized nano-oxides finely and homogeneously dispersed within the matrix.

Figure 9c shows that thermal aging at 500 °C is not able to cause the coarsening of the nano-oxides owing to the sluggish atomic exchanges. However, the increase in solute concentration produced at the interface by the recoil resolution combined with the radiation-enhanced diffusion increases the atomic flux, meaning that solute atoms are transported from one nano-oxide to another [3]. Therefore, the thermal process combined with the irradiation effect is likely to trigger particle coarsening at 500 °C. Figure 3c, which shows the nano-oxide distribution after 150 dpa, confirms this trend. In this TEM bright-field image, it can be observed that nano-oxides are larger and less numerous than before irradiation (Figure 3a). Their average size is estimated at 5.6 nm, while their density is 1.2 ± 0.4 × 10^22^ m^−3^. By comparing the histogram before (Figure 3d) and after irradiation (Figure 3f), it is observed that irradiation induced a shift of the distribution toward the larger diameter accompanied with the disappearing of the smallest nano-oxides; i.e., particle coarsening occurred. In addition, one may notice that the nano-oxide distribution after irradiation at 500 °C (Figure 3f) is similar to the nano-oxide distribution after thermal aging at 1300 °C over 1 h (Figure 3e). Further, the shape of the nano-oxide has also evolved from a sphere to near-cubical shape. Table 6 resumes the results obtained after irradiation conducted on both Fe-14Cr ODS [9] and Fe-18Cr ODS [7] at various damage doses.

**Figure 13:**
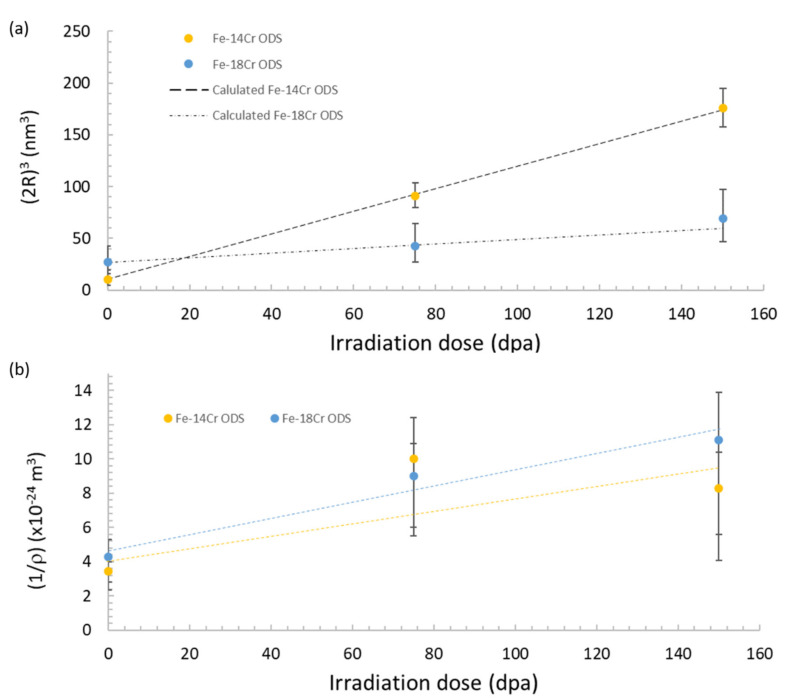
Radius and density evolution after 0, 75 and 150 dpa in both Fe-14Cr ODS and Fe-18Cr ODS: (**a**) evolution of the cube diameter against irradiation damage, (**b**) evolution of the inverse of the density against irradiation damage.

In order to verify if the coarsening regime corresponds to an Ostwald ripening process, the cube radius $R^{3}$ is plotted against the irradiation dose (i.e., the irradiation time multiplied by the dose rate) in Figure 13a. One may observe a linear progression between $R^{3}$ and the irradiation time for both materials. However, the linear progression of the inverse of the density with irradiation time (Figure 13b) is not so clear, especially for Fe-14Cr ODS. For Fe-18Cr ODS, the linear progression of the inverse of the density with the irradiation time appears more obvious considering the error bars. 

Therefore, since the process kinetics scales as $t^{1/3}$, Ostwald ripening could be considered as the mechanism that led the nano-oxides to coarsen, as already suggested in [7], but more data are required to validate this. 

As performed for coarsening during thermal aging, we can use Equation (10) to fit the experimental values and to determine the rate coefficient $K_{irr}\left(T\right)$ of the coarsening process under irradiation:

(10) $$R_{irr}^{3}\left(t\right)-R_{irr}^{3}\left(0\right)=K_{irr}\left(T\right)t$$

The values obtained for the rate coefficient and the calculated values of the nano-oxide radii are given in Table 7 for both the Fe-14Cr ODS and Fe-18Cr ODS steels. Figure 13a proposes a comparison between experimental and calculated values. All the calculated values are in good accordance with the experimental values, except the diameter value after 150 dpa in the Fe-18Cr ODS, which is slightly underestimated. 

By comparing the rate coefficients describing the Ostwald ripening process under thermal (Table 4) and irradiation (Table 7) conditions, one may conclude that for the Fe-14Cr ODS steel, irradiation at 500 °C with a flux of 6.4 dpa·s^−1^ is close to thermal annealing performed at a temperature ranging between 1250 and 1300 °C. The difference in the coarsening rate kinetics observed between Fe-14Cr ODS and Fe-18Cr ODS remains unexplained; the slight difference in the initial nano-oxide size could possibly explain it because, as discussed below, the nano-oxide interface can remove the defect created by irradiation and decrease the diffusion coefficient. Further, difference in matrix composition can also modify the element diffusivity. One may also consider that the nano-oxides are initially non-stoichiometric compounds [7]; this particularity may affect the growth kinetics under irradiation. However, it has been proven that irradiation tends to make the nano-oxides increasingly stoichiometric since the ratio Y:Ti is close to 1 after 150 dpa at 500 °C [7].

#### 3.4.3. Irradiation Response of the Nano-Oxides at Medium Temperature

1. The temperature of apparent stability

At low temperature (i.e., RT), the nano-oxides are unstable as they dissolve, while at high temperature (500 °C), the nano-oxides coarsen. This result means that for a critical temperature, an intermediate behaviour should exist that consists of an apparent stability of the nano-oxides. The apparent stability [33] refers to a regime under which the nano-oxides undergo the ballistic ejection of their constituting atoms but, owing to diffusion, the ejected atom back-diffuses toward its original particle or diffuses toward another particle, where it replaces another constituting atom that had previously been ejected. It can be resumed as a regime where the atomic ejection flux and the atomic arrival flux compensate each other, giving an impression of stability. To reach this intermediate regime, two additional ion irradiations were performed at 300 °C and 400 °C.

**Figure 14:**
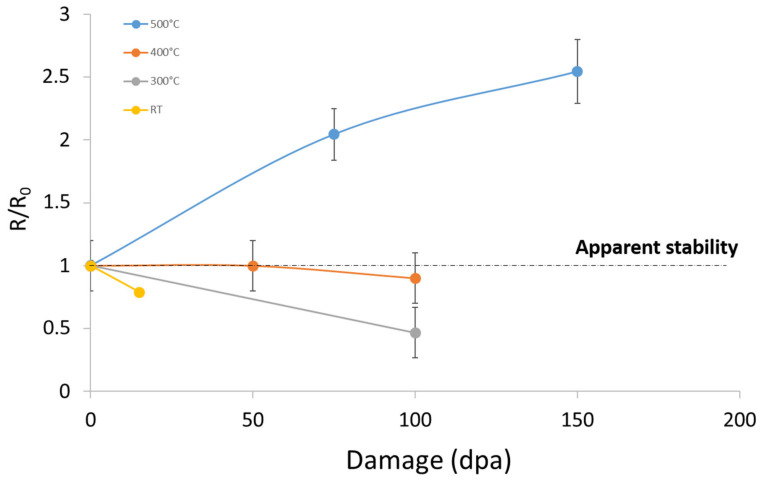
Evolution of the nano-oxides’ normalized radii after irradiation at RT, 300 °C, 400 °C and 500 °C.

Unlike irradiation at 500 °C, the main difficulty for this study resides in the fact that the nano-oxides remain at a very low size and are not easily distinguishable due to the high density of irradiation loops. However, TEM observations allow qualitative results on the few distinguished oxides to be provided. The emerging trend after irradiation at 300 °C is the dissolution of the nano-oxides with a rate slower than the rate corresponding to irradiation at RT (Figure 14). The emerging trend after irradiation at 400 °C is still the dissolution of the nano-oxides, but with an even slower rate (Figure 14). 

Since the data were collected from both Fe-18Cr ODS and Fe-14Cr ODS, the initial average size ($R_{0}$) of the particles is different for each irradiation. In Figure 14, we propose to plot the normalized radius evolution against the irradiation damage at RT, 300 °C, 400 °C and 500 °C. It can be inferred that the temperature of apparent stability for these ODS steels is nearly 400 °C. Even though these results can certainly be improved with a more accurate experimental process, it can be concluded that at this temperature, the nano-oxide dispersion of the ODS steels is preserved. 

Other authors observed a stability of the nano-oxides but at different temperatures. He et al. [4] reported no significant change in the size and number density of the nano-oxide distribution at both 500 °C and 600 °C after Ni^2+^ ion irradiation up to 100 dpa. In the 14YWT ODS steels irradiated up to 100 dpa at 450 °C and 600 °C, the TEM observation performed by Certain et al. [34] revealed a nano-oxide population that was undistinguishable from that of the unirradiated sample.

## 4. Discussion

In this paper, we demonstrated that irradiation can affect the nano-oxide dispersion in ODS steels. When subjected to ion bombardment, the nano-dispersion dissolves at low temperature; in contrast, the oxides coarsen at high temperature. The coarsening mechanism is supposed to be radiation-enhanced Ostwald ripening, where the excess of point defects created by irradiation enhances the thermal diffusion and the ballistic jumps assist the thermal Gibbs–Thomson effect to increase the interfacial solute concentration. Further, the larger oxide tends to dissolve to the benefit of the smaller ones through an inverse Ostwald ripening mechanism. In the end, when thermal and ballistic atomic fluxes compensate each other, the nano-oxides exhibit an apparent stability. Therefore, except at low temperature where the nano-oxides are likely to dissolve completely after a long irradiation, it can be concluded that the nanoprecipitation is generally preserved for the irradiation condition presented in this paper since the nano-oxide average diameter reached a value of only 5.6 nm after the longest irradiation (150 dpa at 500°C). 

Now we know that the nanosize of the oxides is retained under irradiation, there is a functionality that needs to be highlighted: nano-oxides are supposed to act as sinks for point defects [35,36]. This means that point defects created under irradiation can be lost at the nano-oxide interfaces. We use the result obtained in this paper to support this point. To do this, we need to calculate the sink strength of the ODS steel. 

First, we have to calculate the diffusion coefficient under irradiation. For this, we consider that the Ostwald ripening under irradiation is close to the classic Ostwald ripening regime since we proved the coarsening kinetic scale to be $t^{1/3}$. Therefore, we can deduce an equivalent irradiation temperature $T^{eq}\;$by assuming

(11) $$K_{irr}\left(T^{irr}\right)=K_{th}\left(T^{eq}\right)$$

which can be formulated after simplification as

(12) $$\frac{D^{th}C_{\infty }}{T^{eq}}=\frac{D^{irr}C_{\infty }^{irr}}{T^{irr}}$$

**Figure 15:**
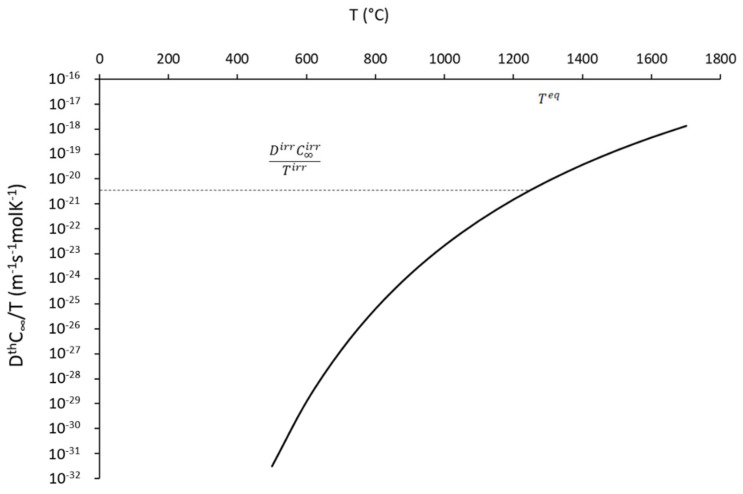
Evolution of $\frac{D^{th}C_{\infty }}{T}$ against temperature, used in order to determine the equivalent temperature.

This equation has to be considered as a first approximation to rationalize the radiation-enhanced coarsening, as explained in [37]. This gives some clues to understand key parameters controlling coarsening under irradiation. From Table 7, it can be seen that $\frac{D^{irr}C_{\infty }^{irr}}{T^{irr}}$ is equal to 5.26 × 10^−21^ m^−1^s^−1^molK^−1^. Figure 15 is the plot of $\frac{D^{th}C_{\infty }}{T}\;$against the temperature. The plot has been realized by using the theoretical value of the diffusion coefficient [23] and solubility [24], as previously given in Table 2. By reporting on this plot the value found for $\frac{D^{irr}C_{\infty }^{irr}}{T^{irr}}$, we can deduce the equivalent annealing temperature; that is, the temperature that would have produced the same result as irradiation but in pure thermal annealing conditions. We found that an irradiation at 500 °C for 9.5 h (i.e., 150 dpa) is equivalent to a thermal annealing at 1270 °C for the same duration. This theoretical result appears to be in good accordance with the theoretical results presented in Figure 9c, showing that a thermal annealing of 10 h at 1270 °C leads the nano-oxides to grow from a radius of 1.4 nm to a radius of about 3 nm. Therefore, if we assume that $C_{\infty }^{irr}\left(T^{irr}\right)=C_{\infty }\left(1270\;{}^{\circ}C\right)$, we can calculate the diffusion coefficient of the rate-controlling element for this irradiation condition; we found a value of $D^{irr}$= 8.5 × 10^−14^ m^2^s^−1^. 

From a theoretical point of view, irradiation enhances the diffusion coefficient $D^{irr}$ relative to the purely thermal valued $D^{th}$ by [38]

(13) $$D^{irr}=\frac{\left(C_{v}^{irr}+C_{v}^{eq}\right)}{C_{v}^{eq}}D^{th}\approx \frac{C_{v}^{irr}}{C_{v}^{eq}}D^{th}$$

where $C_{v}^{irr}$ is the concentration of vacancies under irradiation, and $C_{v}^{eq}$ is the equilibrium concentration of vacancies under purely thermal conditions. In ODS steels, the sink density is high enough to consider that interstitials find the sink before they find vacancies [39]. Therefore, we can approximate the concentration of vacancies as

(14) $$C_{v}^{irr}=\varphi \tau$$

where φ is the defect production equal to a rate of 6.4 × 10^−3^ dpa·s^−1^ [7] and τ is the time for a vacancy to reach a sink. Therefore, after the substitution of Equation (14) into Equation (13), the time τ is equal to [7]

(15) $$\tau =\frac{C_{v}^{eq}}{\varphi }\frac{D^{irr}}{D^{th}}=\frac{e^{-\frac{E_{f}}{kT}}}{\varphi }\frac{D^{irr}}{D^{th}}=\frac{1}{k^{2}D_{v}}$$

With $E_{f}$—the vacancy formation energy—equal to 2.2 eV [40], $k^{2}$ is the global sink strength and $D_{v}$ is the vacancy diffusion coefficient. After calculating $D^{th}$ at 50 0°C from the values given in Table 2, we found that τ is equal to 1.2 × 10^−6^ s. After calculating the vacancy diffusion coefficient $D_{v}$ as proposed in [7], the corresponding global sink strength $k^{2}$ is found to be equal to 3.8 × 10^16^ m^−2^. 

Considering that nano-oxide dispersion, dislocations, free surfaces and grains are the main sinks for point defects [41], we can try to estimate which of these sinks is the principal contributor to the loss of point defects. The sink strength $k_{NO}^{2}$ for the nano-oxides (NO) can be calculated as $k_{NO}^{2}=4\pi R\rho Y$ [41], where R and ρ are the radius and number density of the nano-oxides and *Y* is a constant related to the bias for defect trapping [41], which is reported to be equal to 1 [41]. The sink strength $k_{NO}^{2}$ is estimated by considering the average radius and number density from both TEM (this study) and APT measurement [42]. The sink strength for the dislocations can be calculated as $k_{disl}^{2}=Z_{d}\rho_{d}$, where $Z_{d}$ is the atomic site for defect capture according to the dislocation line intercepted with a crystal plane [41] and $\rho_{d}$ is the dislocation density. For the calculation, we approximate the dislocation sink strength as being similar to the dislocation density [41]. The dislocation density was estimated to be 5.0 × 10^14^ m^−2^ in similar ferritic ODS steels [28]. The sink strength of the free surfaces in thin foil can be calculated as [43] $k_{s}^{2}=3/l^{2}$ by considering that $\underset{i}{\sum }k_{i}l\to 0$, where $\underset{i}{\sum }k_{i}$ is the sink strength of all other microstructural defect sinks within the foil and *l* is the half of the foil thickness, estimated at 50 nm. Concerning the grain, the sink strength can be expressed as $k_{gb}^{2}=60/d^{2}$ by considering that $\underset{i}{\sum }k_{i}d\to 0$ [41], with *d* being the diameter of the grains, which is equal to 0.5 µm in the studied ODS steels [44]. Table 8 presents the result for all the sink strength.

The sum of all the sink strengths $k^{2}=k_{NO}^{2}+k_{disl}^{2}+k_{s}^{2}+k_{gb}^{2}$ is equal to 5.9 × 10^15^ m^−2^ when taking into account $k_{NO}^{2}$ (TEM) and is equal to 1.8 × 10^16^ m^−2^ when taking into account $k_{NO}^{2}$ (APT). The sink strength calculated based on the APT measurement matches the sink strength value found previously (3.8 × 10^16^ m^−2^), which validates this method over the TEM measurement. Thus, as Aydogan et al. [41] found, one may note that $k_{NO}^{2}>k_{s}^{2}>k_{disl}^{2}>k_{gb}^{2}$, with $k_{NO}^{2}$ one order of magnitude higher than the other sink strength. Hence, it can be concluded that the nano-oxides are likely to be the main mechanism of defect removal under irradiation.

From this sink calculation, the removal of point defects by the sample surface appears to be significant. Since thin foil geometry was used to irradiate the ODS samples, the loss of point defects at the free surface should modify the kinetics of growth in the case where the thermal effects predominate over the ballistic effects. Thus, at 500 °C, the growth of the nano-oxides may be over or underestimated due to the effect of free surfaces. 

In conclusion, in addition of being obstacles to the dislocation motion, nano-oxides also act as sinks for the point defects created under irradiation and allow their population to be reduced. This functionality requires the stability of the atomic configuration of the interfaces, but recent results [12] showed that interfaces of nano-oxides exhibit instabilities only for high irradiation damage while remaining stable otherwise.

## 5. Conclusions

ODS steels are nanostructured materials as they are reinforced by nano-sized oxides dispersed within the ferritic matrix. The nano-oxides serve as obstacles for the dislocation motion and then prevent the alloys from creep deformation. However, since ODS alloys are meant to be used in harsh environments, nano-oxides must remain stable under irradiation. In this paper, we first show that the nano-oxides exhibit a coarsening resistance since a temperature at least equal to 1100 °C must be applied to trigger the Ostwald ripening process. Under irradiation, the behaviour of the nano-oxides is a function of the temperature. At low temperature, the sluggish nature of the diffusion makes the ballistic ejection the main process for atoms to move and consequently leads to the dissolution of the nano-oxides. At medium temperature, the thermal diffusion compensates the ballistic jumps, giving an impression of apparent stability: the solute atoms no longer remain in the matrix but re-precipitate in the dissolving oxide or in a neighbour particle. At high temperature, the thermal effects dominate the ballistic exchanges, and the nano-oxides start to coarsen. Ostwald ripening is enhanced by the point defects that favour atomic exchanges between particles, but also by the ballistic ejection that enhances the Gibbs–Thomson effect. Ostwald ripening can also be inverted and conducts the large nano-oxides to dissolve to the benefit of the smallest ones. However, the coarsening kinetics are low enough to sustain the nano-oxides at a nanometric scale. In the end, the nano-oxides exhibit another functionality as they appear to be the main sink that is able to remove vacancies and interstitials created during irradiation significantly.

## Figures and Tables

**Table 1:** Chemical compositions (wt%) of the studied materials: Fe-14Cr ODS and Fe-18Cr ODS.

Cr	W	Ti	Mn	Si	Ni	C	Y_2_O_3_	Fe
14	1	0.3	0.3	0.3	0.15	0.05	0.3	Bal.
18.05	0.95	0.26	0.3	0.3	0.19	0.03	0.56	Bal.

**Table 2:** Values used to calculate the solute concentration according to Equation (4). $D_{0}$ is the Y diffusion pre-exponential factor, $E_{m}$ is the diffusion activation energy of Y, $C_{\infty }$ is the equilibrium solubility of Y in the α-Fe matrix, $E_{s}$ is the equilibrium solubility activation energy of Y, qϕ is the irradiation damage rate, λ is the mean displacement of Y ejected atoms, γ is the interface energy of Y_2_Ti_2_O_7_ nano-oxide and $V_{m}$ is the Y_2_Ti_2_O_7_ molar volume.

$D_{0}\;(m^{2}\cdot s^{-1})$	$E_{m}\;(\mathrm{eV})$	$C_{\infty }\;(\mathrm{mol}\cdot m^{-3})$	$E_{s}\;(\mathrm{eV})$	$q\phi \;(\mathrm{dpa}\cdot s^{-1})$	λ (nm)	γ	$V_{m}\;(m^{3}\cdot {\mathrm{mol}}^{-1})$
5.7 × 10^−7^ [23]	2 [23]	5.4 [24]	1.5 [24]	6.5 × 10^−3^ [7]	0.35 [7]	0.26 [25]	7.71 × 10^−5^ [24]

**Table 3:** Radius evolution with standard deviation s at 1250 °C, 1300 °C and 1400 °C from [28] and [29]. Adapted from [28] with permission from Elsevier.

*T* (°C)	*t* (h)	*R* (nm)	*s*	*R* (nm) TEM
1250	0	1.4 [28]	0.5 [28]	1.3 [28]
	1	1.6 [28]	0.5 [28]	
1300	0	1.4 [28]	0.5 [28]	1.3 [28]
	1	2.0 [28]	0.7 [28]	
	3	2.6 [28]	0.9 [28]	
1400	0	1.4 [28]	0.5 [28]	1.3 [28]
	1	3.0 [28]	0.9 [28]	2.4 [28]
	3	4.4 [29]	0.03 [29]	

**Table 4:** Rate coefficient $K_{th}\left(T\right)$ for p = 3 at various temperatures.

*T* (°C)	$K_{th}\left(T\right)\;\times \;10^{-3}\;({\mathrm{nm}}^{3}\cdot s^{-1})$
1250	0.7
1300	1.6
1400	7.5

**Table 5:** Parameters of the modified Frost and Russell model fitted to the experimental data.

$2R_{0}(\mathrm{nm})$	L	$S_{0}(\mathrm{dpa}\cdot s^{-1})$	$\lambda_{m}(\mathrm{nm})$	$R_{m}(\mathrm{nm})$	B
14.4	50	9.9 × 10^−4^	7	5.7	0.2

**Table 6:** Mean diameter and density of nano-oxides after ion irradiation in both Fe-14Cr ODS and Fe-18Cr ODS [7,9].

	Damage Dose (dpa)	Mean Diameter (nm)	Density (m^−3^)
Fe-14Cr ODS [9]	0	2.2 (±0.5)	2.9 ± 0.5 × 10^23^
	75	4.5 (±0.2)	1 ± 0.3 × 10^23^
	150	5.6 (±0.2)	1.2 ± 0.4 × 10^22^
			
Fe-18Cr ODS [7]	0	3.0 (±0.5)	2.3 ± 0.7 × 10^23^
	75	3.5 (±0.5)	1.1 ± 0.3 × 10^23^
	150	4.1 (±0.5)	0.9 ± 0.3 × 10^23^

**Table 7:** Comparison between experimental and calculated nano-oxide diameter after radiation-enhanced Ostwald ripening in (a) Fe-14Cr ODS steel and (b) Fe-18Cr ODS steel.

(a)			
*K_irr_*(*T*) × 10^−3^ (nm^3^.s^−1^)	Damage dose (dpa)	Experimental diameter 2R (nm)	Calculated diameter 2R (nm)
0.87	0	2.2 (±0.5)	-
	75	4.50 (±0.2)	4.52
	150	5.60 (±0.2)	5.58
(b)			
*K_irr_*(*T*) × 10^−3^ (nm^3^.s^−1^)	Damage dose (dpa)	Experimental diameter 2R (nm)	Calculated diameter 2R (nm)
0.17	0	3.0 (±0.5)	-
	75	3.5 (±0.5)	3.51
	150	4.1 (±0.5)	3.90

**Table 8:** Sink strength values for nano-oxides, dislocations, free surface and grains.

$k_{NO}^{2}\;(\mathrm{TEM})\;(m^{-2})$	$k_{NO}^{2}\;(\mathrm{APT})\;(mm^{-2})$	$k_{disl}^{2}\;(m^{-2})$	$k_{s}^{2}\;(m^{-2})$	$k_{gb}^{2}\;(m^{-2})$
4.0 × 10^15^	1.6 × 10^16^	5.0 × 10^14^	1.2 × 10^15^	2.4 × 10^14^

## Data Availability

The data presented in this study are available on request from the corresponding author. The data are not publicly available due to privacy property.
